# Mechanism, Kinetics and Thermodynamics of Decomposition for High Energy Derivatives of [1,2,4]Triazolo[4,3-*b*][1,2,4,5]tetrazine

**DOI:** 10.3390/molecules27206966

**Published:** 2022-10-17

**Authors:** Aleksandr V. Stankevich, Svetlana G. Tolshchina, Anna V. Korotina, Gennady L. Rusinov, Irina V. Chemagina, Valery N. Charushin

**Affiliations:** 1 I.Ya. Postovsky Institute of Organic Synthesis, Ural Branch of the Russian Academy of Sciences, S. Kovalevskaya Str., 22/20, 620108 Ekaterinburg, Russia; 2 Russian Federal Nuclear Center, All-Russian Research Institute of Technical Physics (RFNC-VNIITF), Vasilieva Street 13, 456770 Snezhinsk, Russia

**Keywords:** energetic materials, tetrazines, thermal decomposition, kinetics, decomposition reaction products

## Abstract

This paper presents the data of research studies on the mechanisms, kinetics and thermodynamics of decomposition of three high-energy compounds: [1,2,4]triazolo[4,3-*b*][1,2,4,5]tetrazine-3,6-diamine (TTDA), 3-amino-6-hydrazino[1,2,4]triazolo[4,3-*b*][1,2,4,5]tetrazine (TTGA) and 3,6-dinitroamino[1,2,4]triazolo[4,3-*b*][1,2,4,5]tetrazine (DNTT). The points of change of the reaction mechanisms under thermal effects with different intensities from 0.1 to 2000 s^−1^ have been established. The values of activation and induction energies for the limiting stages of decomposition have been obtained. The formation of nanostructured carbon nitride (α-C_3_N_4_) in condensed decomposition products, cyanogen (C_2_N_2_) and hydrogen cyanide (HCN) in gaseous products have been shown. Concentration-energy diagrams for the reaction products have been compiled. The parameters of heat resistance and thermal safety proved to be: 349.5 °C and 358.2 °C for TTDA; 190.3 °C and 198.0 °C for TTGA; 113.4 °C and 114.1 °C for DNTT. The energy and thermodynamic properties have also been estimated. This work found the activation energy of the decomposition process to be 129.0 kJ/mol for TTDA, 212.2 kJ/mol for TTGA and 292.2 kJ/mol for DNTT. The average induction energy of the catalytic process (Ecat) for TTGA was established to be 21 kJ/mol, and for DNTT-1500–1700 kJ/mol. The induction energy of the inhibition process (Eing) of TTDA was estimated to be 800–1400 kJ/mol.

## 1. Introduction

**Scheme 1:**
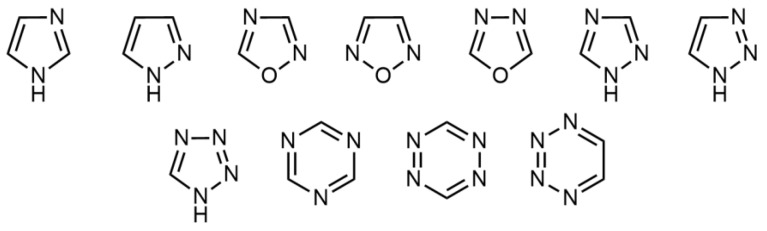
Chemical structures of azoles and azines commonly employed in high-energy heterocyclic compounds.

High-energy compounds (HECs) are used in many areas of civil industry, such as the synthesis of superhard materials like diamond and ceramics, in car airbags, in mining, recycling of waste rubber products and in other areas. At the same time, the sensitivity of HEC to external influences limits scenarios of their use in various fields of technology. In this regard, the search for insensitive HECs remains an urgent task. Many creative teams have demonstrated in their works that polynitrogen-containing heterocyclic compounds, such as nitro-, amino- and nitroamino-derivatives of imidazole, pyrazole, oxadiazoles, triazoles, tetrazole, triazines and tetrazines (Scheme 1) and condensed structures based on these heterocycles, have the required balance of energy properties and sensitivity to external influences [1,2,3,4,5,6,7,8,9,10,11,12]. The experimental data obtained indicate the expediency of performing exploratory work, and analyzing the mechanisms of decomposition of polynitrogen heterocyclic compounds, which can primarily be focused on the synthesis of promising materials, for example, polymer ceramics. In this field, derivatives of [1,2,4]triazolo[4,3-*b*][1,2,4,5]tetrazine [13,14,15,16,17,18,19,20,21] can find a practical application as HECs; however, the formation of volatile cyanides during decomposition of these compounds raises concerns regarding their toxicological safety.

Some derivatives of [1,2,4]triazolo[4,3-*b*][1,2,4,5]tetrazine-3,6-diamine [13,14,15] are of interest as tetrazines having a high temperature resistance, and hence safety. In addition, due to the relative simplicity of this molecular structure, it is advisable to use this compound as a model, when forming an initial approximation of experimental data for decomposition processes in triazole and tetrazine derivatives. In this paper, derivatives of [1,2,4]triazolo[4,3-*b*][1,2,4,5]tetrazine-3,6-diamine are considered as starting materials for the synthesis of polymer ceramics based on carbon nitride (C_3_N_4_) [22,23,24,25,26,27,28].

**Scheme 2:**
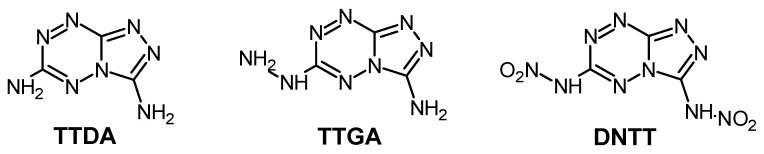
Chemical structures of the studied triazolo[4,3-*b*][1,2,4,5]tetrazine derivatives.

[1,2,4]Triazolo[4,3-*b*][1,2,4,5]tetrazine-3,6-diamine (TTDA) [29] and its two analogues 3-amino-6-hydrazino[1,2,4]triazolo[4,3-*b*][1,2,4,5]tetrazine (TTGA) [18] and 3,6-dinitroamino[1,2,4]triazolo[4,3-*b*][1,2,4,5]tetrazine (DNTT) [13] (Scheme 2) were selected for further study of their properties and decomposition processes.

The characteristics of some compounds presented are described in the literature (Table 1).

On the one hand, the mechanisms and directions of chemical reactions are the main physico-chemical characteristics for HECs. On the other hand, experimental studies aimed at carrying out such measurements have many technical and fundamental limitations. Therefore, the problems of constructing energy diagrams of slow decay and explosive transformation processes directly from the data of experimental observations require non-trivial solutions. In order to identify the energy potential and regulate the energy release in the considered range of substances, research studies of chemical reactions occurring under various thermal conditions have been carried out.

## 2. Results

The frequency, structure and elemental composition of the synthesized compounds were confirmed by ^1^H and ^13^C NMR, IR and RAMAN spectroscopy, chromato-mass spectrometry and elemental analysis. The crystal structure was determined by powder X-ray diffraction methods. The monophase of the sample was confirmed by X-ray phase analysis and Raman spectroscopy.

### 2.1. Thermodynamics Calculation Results

For the substances under consideration, thermodynamic calculations were carried out using classical methods [31,32,33,34,35] and semi-empirical methods of quantum chemistry.

Table 2 shows the values obtained from thermodynamic calculations.

From the results obtained, it can be assumed that the expected density in calculations by the group contribution method is close to real substances. The molecular volume method shows highly inflated results and does not take into account the packing coefficient. In addition, it is worth noting that each of the polymorphic modifications will have its own density values.

**Figure 1:**
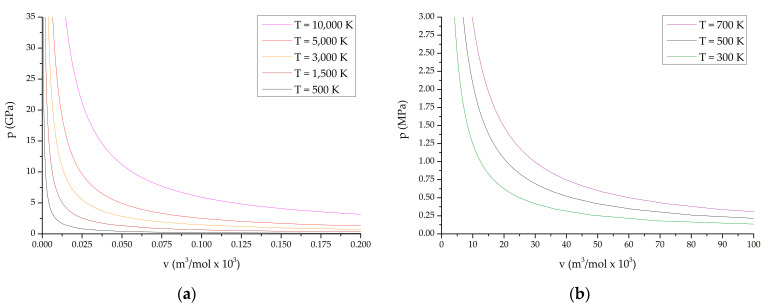

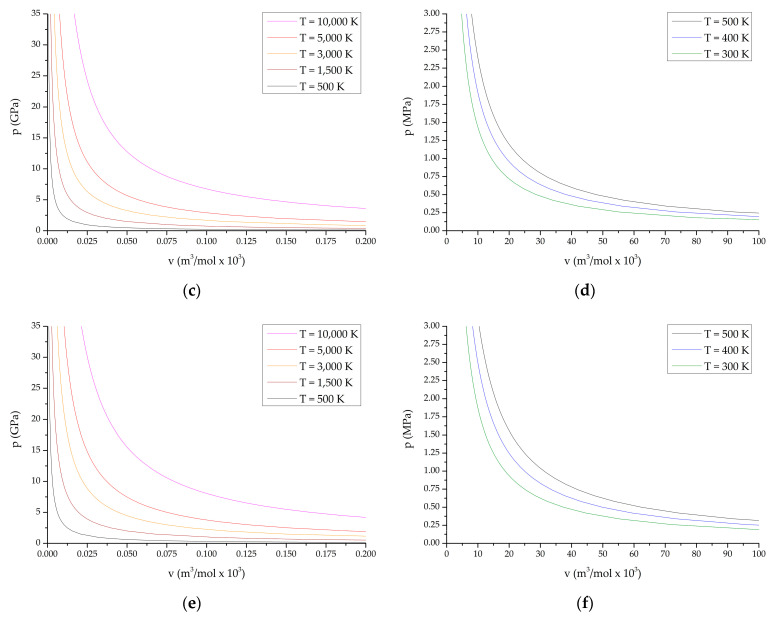
P-V diagrams of reaction products from the state of compressed cold plasma to normal temperature: (**a**,**b**) TTDA; (**c**,**d**) TTGA; (**e**,**f**) DNTT.

Below, Figure 1 shows the obtained PV diagrams of decomposition products of the studied HEC. The diagrams (Figure 1) characterize various processes and directions of chemical reactions during decomposition of the compounds under study. In addition, studies of the composition of reaction products under various conditions have been carried out.

P-V diagrams of reaction products for explosive transformation processes (deflagration, explosive burning, normal detonation and recompressed detonation) are shown in Figure 1a,c,e; P-V diagrams of reaction products for slow thermal decomposition and combustion processes are shown in Figure 1b,d,f. The course of reactions in slow thermal decomposition modes of decomposition and combustion is characteristic of the conditions realized in the cells of thermal analysis at heating rates up to the limit values of the thermal stability of the substance under study.

The purpose of the experimental part of this work is to determine substances’ decomposition temperatures under various conditions and the kinetics and their heat resistance, with the subsequent connection of the obtained values and reaction products to theoretical models.

### 2.2. Structure and Chemical Composition of Synthesized Substances

[1,2,4]Triazolo[4,3-*b*][1,2,4,5]tetrazine-3,6-diamine (TTDA) was synthesized by a well-known technique [29,36].

**Figure 2:**
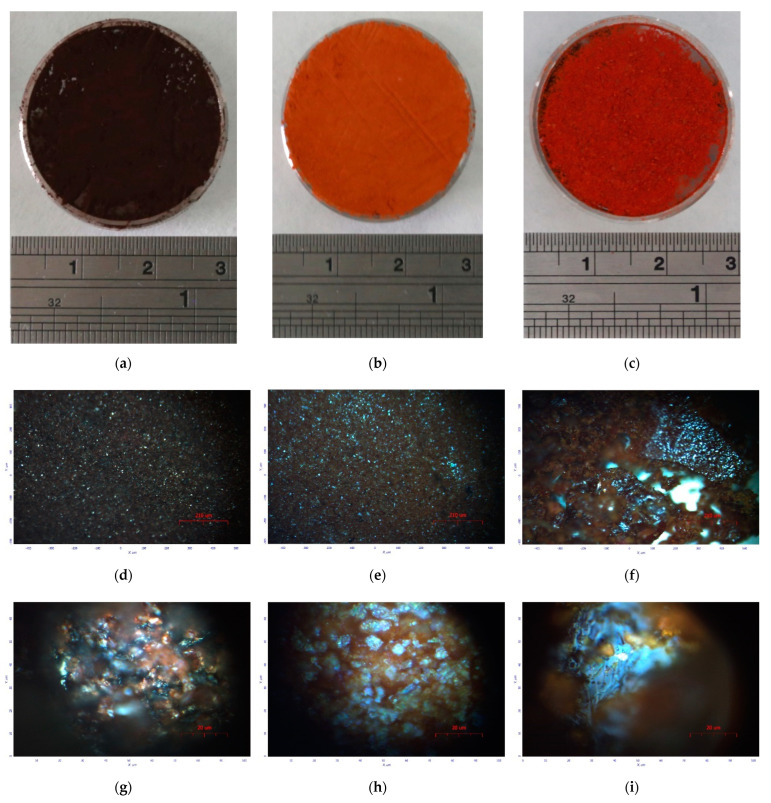
Appearance of synthesized samples and micromorphology of particles: (**a**,**d**,**g**) TTDA; (**b**,**e**,**h**) TTGA; (**c**,**f**,**i**) DNTT.

The characteristics determined in our study were as follows: 

Dark brown powder (Figure 2a,d,g). ^1^H NMR (DMSO-d_6_, 500 MHz): δ/ppm 6.82, 7.60 (both s, 4H, 2 NH_2_). ^13^C NMR (DMSO-d_6_, 125 MHz): δ/ppm 148.3, 148.6, 155.2 (the NMR data are consistent with the literature [29]). LC/MS (ESI): Found *m*/*z* = 151.0485 (M − H). C_3_H_3_N_8_^-^. Calculated 151.0486 (M − H).

3-Amino-6-hydrazino[1,2,4]triazolo[4,3-*b*][1,2,4,5]tetrazine (TTGA) was synthesized by a well-known method [18].

Brown powder (Figure 2b,e,h). ^1^H NMR (DMSO-d_6_, 500 MHz): δ/ppm 4.39 (br s, 2 H, NH_2_); 6.93 (s, 2 H, NH_2_); 9.61 (br s, 1 H, NH). ^13^C NMR (DMSO-d_6_, 125 MHz): δ/ppm 148.8, 148.9, 156.5 (the NMR data are consistent with literature [18]). LC/MS (ESI): Found *m/z* = 166.0594 (M − H). C_3_H_4_N_9_^-^. Calculated 166.0595 (M − H).

3,6-Dinitroamino[1,2,4]triazolo[4,3-*b*][1,2,4,5]tetrazine (DNTT) was synthesized by a well-known method [13].

Orange powder (Figure 2c,f,i). ^1^H NMR (DMSO-d_6_, 500 MHz): δ/ppm 11.97 (br s, 2 H, 2 NH). ^13^C NMR (DMSO-d_6_, 125 MHz): δ/ppm 143.1, 144.2, 156.1 (the NMR data are consistent with literature [13]). LC/MS (ESI): Found *m/z* = 241.0185 (M − H). C_3_HN_10_O_4_^-^. Calculated 241.0188 (M − H).

Spectral studies

TTDA compound found absorption bands, sm^−1^ (T%):

519 (64.7); 528 (58.3); 540 (45.3); 547 (46.4); 553 (45.4); 561.6 (47.8); 571 (55.8); 582 (35.9); 592 (37.5); 604 (43); 620 (34.8); 657 (58.2); 678 (29.7); 707 (46.5); 737 (46.3); 753.5 (29.1); 765 (36.2); 836 (32.3); 872 (37.5); 946 (38.1); 1043 (61.1); 1093 (22.8); 1140.2 (26.6); 1166 (35.1); 1225.5 (25.7); 1262 (41.7); 1273.1 (35.7); 1318 (40.8); 1367 (36.4); 1402 (33.6); 1462 (47.2); 1478 (25.6); 1524.7 (37); 1546 (52.6); 1591.5 (48.5); 1605 (56); 1649 (48.8); 2499 (15.1); 2683 (20.9); 2727 (22.1); 3016.5 (29.5); 3106 (40.1); 3193.2 (31.8); 3208 (32); 3283 (37.4); 3421 (27.3).

TTGA compound found absorption bands, sm^−1^ (T%):

518 (94); 522.9 (84.6); 527.3 (84.4); 531 (84.9); 534.9 (86.8); 547.3 (91); 550.5 (90.2); 555.1 (87.8); 566.7 (82.5); 580 (78.3); 607 (85.3); 619.9 (79.2); 679 (56.7); 726.2 (42.9); 740 (63.9); 758 (39); 766.4 (35.6); 792 (28.8); 893 (41); 967 (47); 1022 (74.6); 1053 (54.9); 1106 (24.7); 1139 (26); 1185 (48.2); 1228.3 (30); 1265 (59.4); 1296 (25.3); 1326 (27.8); 1346.6 (30.5); 1374 (42.6); 1392 (55.8); 1455 (36.1); 1485 (52.1); 1518 (48.5); 1532 (54.7); 1571 (77.3); 1627.7 (71.1); 1643 (83.7); 1703.8 (9.7); 2407.2 (12.8); 2449 (13); 2532 (16); 2719 (19); 2793 (22.5); 2839.3 (25.3); 2868.5 (26.5); 2909 (32); 2976.5 (39.1); 3025 (44.9); 3103 (50.1); 3140.4 (47.2); 3187.7 (46.6); 3236 (49.6); 3297 (46.7); 3323 (37.6); 3594.2 (9.5).

DNTT compound found absorption bands, sm^−1^ (T%):

522.0 (59.95); 530.0 (54.31); 546.0 (55.15); 558.0 (50.62); 566.0 (49.15); 582.0 (46.86); 588.0 (45.95); 609.0 (52.06); 634.0 (52.45); 674.0 (59.89); 687.0 (59.53); 733.0 (52.30); 745.0 (51.11); 770.0 (45.55); 795.0 (35.59); 839.0 (34.77); 868.0 (33.63); 884.21 (33.71); 902.0 (35.16); 976.0 (56.97); 995.0 (40.15); 1010.0 (38.66); 1095.0 (52.07); 1135.61 (39.04); 1147.0 (44.44); 1153.0 (43.88); 1207.0 (64.47)(δNO_2_); 1217.0 (62.12)(δNO_2_); 1253.0 (52.42); 1295.0 (56.01); 1328.0 (39.63); 1369.66 (24.65); 1377.0 (26.06); 1423.0 (28.69); 1443.0 (34.37); 1454.81 (35.35); 1468.93 (39.47); 1481.0 (47.10); 1504.0 (35.11); 1516.35 (29.17); 1536.0(39.14); 1544.40 (30.00); 1573.0 (43.58); 1594.0 (46.00); 1626.0 (34.28)(ν_as_NO_2_); 1638.0 (39.92)(ν_as_NO_2_); 1708.44 (7.54); 2679.0 (19.06); 2758.0 (18.64); 2825.31 (17.07); 2869.0 (16.51); 2908.0 (16.35); 2976.0 (16.96); 3002.0(16.49); 3060.50 (16.52); 3108.0 (17.28); 3249.0 (14.39); 3277.0 (14.60); 3307.0 (13.47).

Thus, from the results of the analysis of the vibrational states of the molecular system, the appearance of substituents and their contribution to the energy of vibrations can be seen.

### 2.3. X-ray Diffraction Studies

The studies were carried out on a laboratory X-ray powder diffractometer in a standard configuration.

X–ray diffraction analysis conditions: pressure 730–740 mmHg, temperature 22–24 °C; source—X-ray tube with copper anode (working radiation of K series: K_α1_, K_α2_ and K_β_ for copper corresponds to wavelengths of 1.5406 Å, 1.5444 Å and 1.3920 Å (working energy of quanta ~8keV); detector-semiconductor with cooling Peltier; geometry—Bregg–Brentano (θ-2θ); scanned angle range=0.8–70 degrees; scanning step=1.214 degrees/minute; accumulation time–1...1.2 s.

TTDA connection found reflection planes, d (Å):

7.208; 6.885; 5.505; 5.334; 4.835; 4.697; 3.911; 3.697; 3.456; 3.342; 3.232; 3.082; 2.995; 2.952; 2.756; 2.716; 2.665; 2.532; 2.46; 2.431; 2.291; 2.281; 2.248; 2.197; 2.059; 2.023; 1.866.

TTGA connection found reflection planes, d (Å):

13.654; 8.009; 6.81; 6.56; 6.254; 5.938; 5.143; 4.976; 4.504; 4.335; 4.091; 4; 3.923; 3.439; 3.382; 3.174; 2.991; 2.956; 2.805; 2.605; 2.455; 2.291.

DNTT connection found reflection planes, d (Å):

10.816; 9.116; 7.351; 5.875; 5.42; 5.013; 4.780; 4.553; 4.132; 3.979; 3.715; 3.683; 3.623; 3.548; 3.497; 3.376; 3.189; 3.100; 3.022; 2.949; 2.827; 2.766; 2.746; 2.708; 2.664; 2.620; 2.612; 2.520; 2.495; 2.443; 2.363; 2.339; 2.304; 2.270; 2.248; 2.202; 2.167; 2.140; 2.120; 2.101; 2.064; 2.024; 1.950; 1.925; 1.884; 1.865; 1.827; 1.818; 1.782; 1.773; 1.718; 1.7; 1.638; 1.615; 1.584; 1.554; 1.534; 1.507; 1.506; 1.471; 1.44; 1.403.

The interplane distances (d) measured by powder X-ray diffraction method, as well as their processing by Le Bail [37] and Pawley [38], made it possible to characterize the crystal lattice of the studied substances. Table 3 shows the results of the analysis.

The solution of the crystal structure of the samples was performed by sequential analysis of radiographs. Determination of interplanar distances was performed by the method of full-profile analysis. The accepted initial conditions for calculating X-ray diffractions data are: the crystallographic model is the result of primary X-ray diffraction; the radiation source is an X-ray tube with a copper anode (K_α1_=1.5406 Å), a semiconductor detector, a pseudo-Voigt and Pearson-VII profile function, the simulated range is 2Θ-2-60°, the initial half-width parameter is FWHM 0.01.

Indexing was carried out by the Monte Carlo method and using standard dichotomy algorithms: DICVOL06 [39] and THREOR [40]. Refinement of the unit cell parameters was performed by the methods of Le Bail [37] and Pawley [38], the results are summarized in Table 4. Statistical characteristics of the analysis were entered into the final CIF files. All characteristics of the crystalline compounds, including the electronic structure, were obtained for the first time in this work.

### 2.4. Characterization of Substances According to Derivatography

**Figure 3:**
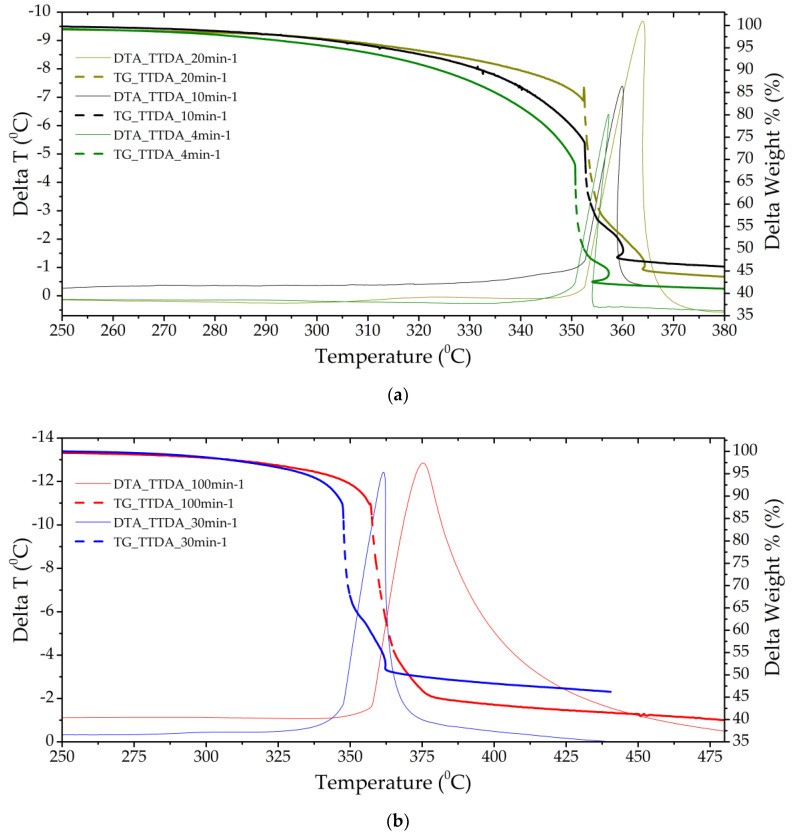
Thermograms of a TTDA sample with a separate stage of gas-phase decomposition (**a**) (heating rates of 4 and 10 min^−1^) and solid-phase decomposition with gas-phase assistance (**b**) (heating rates of 30 and 100 min^−1^).

**Figure 4:**
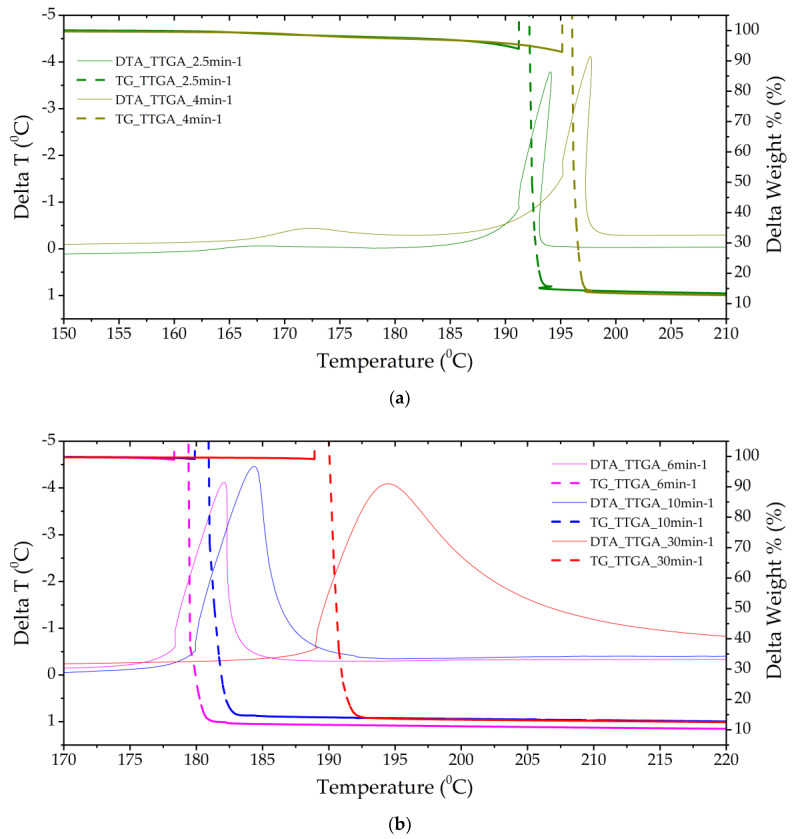
Thermograms of a TTGA sample with a separate stage of gas-phase decomposition (**a**) (heating rates of 2.5 and 4 min^−1^) and solid-phase decomposition with gas-phase assistance (**b**) (heating rates of 6, 10 and 30 min^−1^).

**Figure 5:**
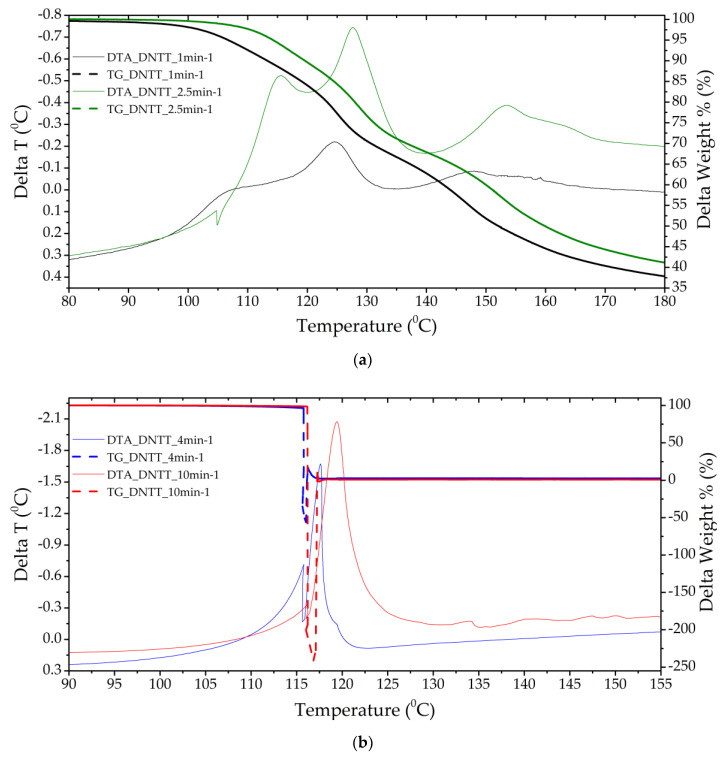
Thermograms of a DNTT sample with a separate stage of gas-phase decomposition (**a**) (heating rate 1 and 2.5 min^−1^) and solid-phase decomposition with gas-phase assisted (**b**) (heating rates 4 and 10 min^−1^).

Studies of the thermal decomposition of [1,2,4]triazolo[4,3-*b*][1,2,4,5]tetrazine derivatives were performed by derivatography methods with synchronous registration of mass loss data using microweights (TGA) and heat flux from the substance using thermocouples (DTA). The studies were carried out as follows: a weight of 5 mg was placed in a ceramic crucible, which was installed in a heating element and heated at various speeds of 2.5, 4, 6, 10, 15, 30 and 100 K/min, with data accumulation statistics 190 points/K, 80 points/K, 48 points/K and 30 points/K, respectively, in the temperature range from 300 to 770 K, at a dynamic flow rate of nitrogen gas, 40.2 cm^3^/min. The results of DTA and TGA are presented in Figure 3, Figure 4 and Figure 5, and in Table 4, Table 5 and Table 6.

Table 4 and Table 5 show the temperature characteristics of the decomposition processes of samples of [1,2,4]triazolo[4,3-*b*][1,2,4,5]tetrazine derivatives at different heating rates. The measurements were obtained by the methods of derivatography: DTA (Table 4), TGA (Table 5).

Data from differential thermogravimetric analysis were used to analyze the points of intensive decomposition. Thermogravimetry results and differential thermocouple readings were used to evaluate the processes occurring without significant loss of mass. Standard indium samples were used as the calibration of the heat flow measurement.

Under thermal exposure to samples of [1,2,4]triazolo[4,3-*b*][1,2,4,5]tetrazine derivatives, we can observe the processes of intensive sublimation, the first stage of decomposition and the second stage of decomposition. Moreover, the processes of the first stage of decomposition, characteristic of slow heating with speeds of less than 3 min^−1^, 6 min^−1^, and 10 min^−1^ for DNTT, TTGA and TTDA, respectively, practically add up to the processes of reactive decomposition. These processes are manifested on thermograms (Figure 3, Figure 4 and Figure 5) in the form of exothermic peaks showing their caloric content. Table 4, Table 5 and Table 6 show the characteristic temperatures for each of the processes obtained as a result of preliminary analysis of thermograms.

We associate the appearance of the first decomposition peaks at slow heating rates with three processes:

1. Decomposition in the gas phase as the most probable process. Moreover, the higher the accumulation of gases, the greater the thermal effect of the reaction. This is due to the ability of substances to sublimate.
2. The first stage of decomposition in the solid phase, due to the separation of substituents from the base of azolotetrazine. This hypothesis arose as a result of the analysis of the magnitude of the shift of the peak of the rate of the solid-phase decomposition process, which is inhibited by the reaction products. In contrast to the generally accepted hypotheses about the catalytic effect of the gas phase of decomposition products, we have recorded inhibition in azolotetrazine compounds.
3. Exothermic phase transition.
4. The presence of impurities similar in molecular structure, but less heat-resistant than the main phase.

**Figure 6:**
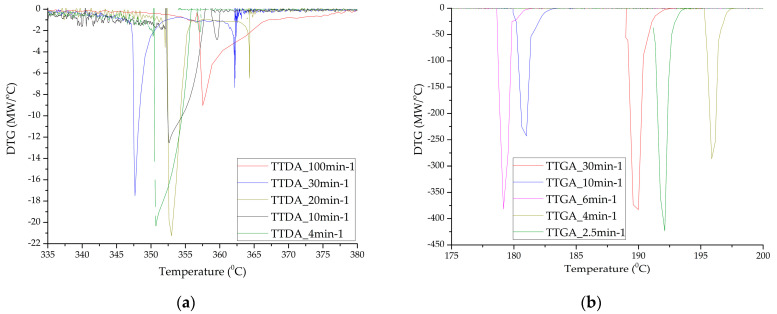

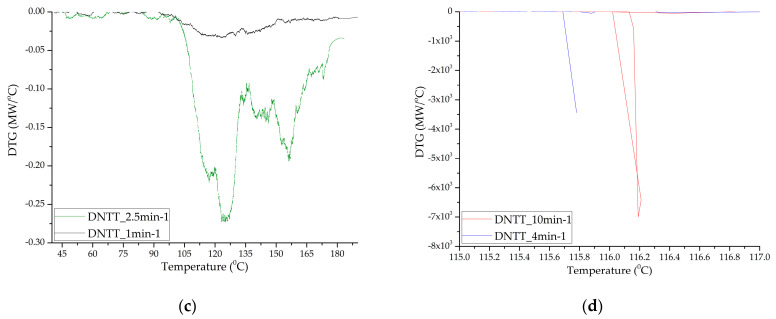
Differential decomposition curves of samples TTDA (**a**); TTGA (**b**) and DNTT (**c**,**d**).

**Figure 7:**
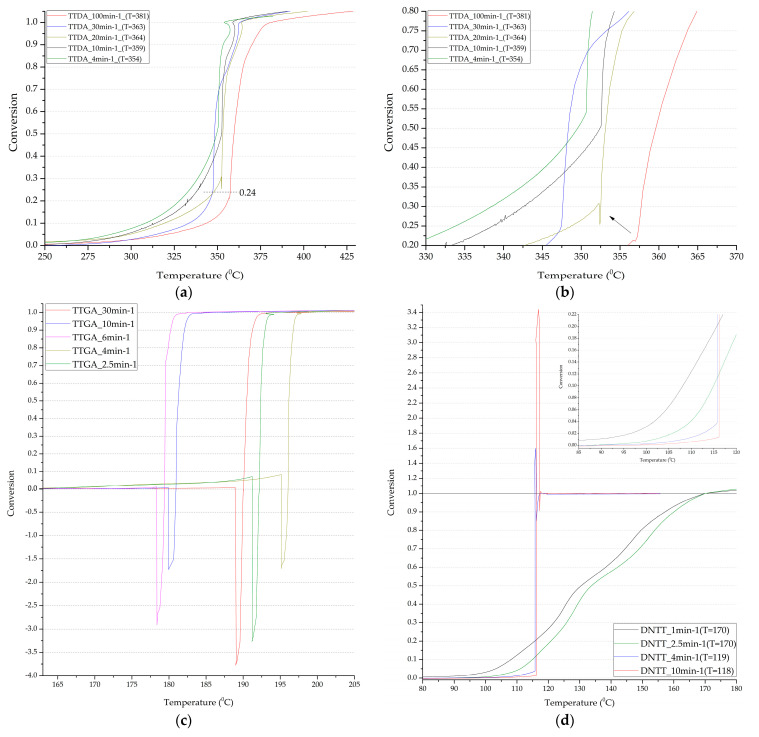
Integral decomposition curves of samples TTDA (**a**,**b**); TTGA (**c**); DNTT (**d**).

These processes can occur both jointly and separately. We divided the contribution of each of the stages, taking into account the parameters of the decomposition kinetics: the activation energy (and adiabatic induction energy) and the rate constant of the chemical reaction, supplemented with data on the composition of the products of the gas phase during the reaction. A more detailed analysis was carried out by the DTGA method (Figure 6) and the construction of integral decomposition curves (Figure 7).

After preliminary data processing, the studied samples can be characterized as follows.

The TTDA sample has a good temperature resistance of ~350 °C, energy release kinetics, residual heat release and low mass loss before decomposition. The mass fraction of condensed products after completion of the decomposition process is more than 40%, which indicates the insufficiency of the oxidizer in the molecule and the purge gas and, possibly, the incomplete course of the decomposition reaction in the main stage (Figure 3).

The TTGA sample showed the greatest reactivity and an increase in pressure in the chamber; it is obvious that the thermal decomposition of this sample proceeds in the explosive combustion mode. However, the momentum of the formed gases does not have high values. The substance has a temperature resistance up to 180 °C (Figure 4). The mass fraction of condensed residues is less than 15%. In the DTA cell, the conditions of reactive action on the sensing element were implemented, characterizing the pulse of gas flows.

Samples of DNTT when decomposed in DTA cells showed the highest caloric content, reactivity and momentum (rapid increase in pressure in the chamber). Clearly, this substance, compared to the others presented for analysis, has the lowest activation energy and induction period of adiabatic decomposition, and the highest adiabatic induction energy and number of simultaneous acts of molecular decay. In the DTA cell, at heating rates of more than 2.5 min^−1^, the substance decomposes in the form of an explosive transformation with the spread of reaction products and the formation of condensed products that are scattered around the cell by the gases released. In this regard, the mass fraction of the condensed residue is not measured precisely (Figure 5 and Figure 6). The substance is characterized by low temperature resistance up to 120°C and thermal stability. The total mass fraction of condensed residues during decomposition in nitrogen could not be recorded. A different picture is observed when the rate of thermal exposure decreases to 1–2.5 min^−1^. Three-stage decomposition is observed, which is accompanied by low energy release and the formation of a large number of condensed reaction products, and conditions of slow thermal decomposition are realized. Table 6 shows the characteristics obtained at a standard heating rate of 10 °C/min.

### 2.5. Characteristics of Gaseous Reaction Products at the Peak of Decomposition

The studies were carried out using mass spectrometry methods with various sample inputs: the gas phase from the differential thermal analysis cell through a pulse valve, the formation of the gas phase in a high-vacuum chamber with various types of ionization (field, laser and spark) with the direct connection of the mass spectrometer channel to the decomposition chamber. The mass spectrometer consisted of an electron-spark ionizer, a quadrupole mass filter and a detector in the form of a Faraday cup.

The speed of registration of mass spectra varied from 10 to 100 Da/s, the number of points of the profile line varied from 250 to 10, according to the registration speeds. The energy of the electron beam in the ionizer varied from 90 eV (focus 70 eV) to 25 eV (focus 15 eV) and complete shutdown of the ionizer.

**Figure 8:**
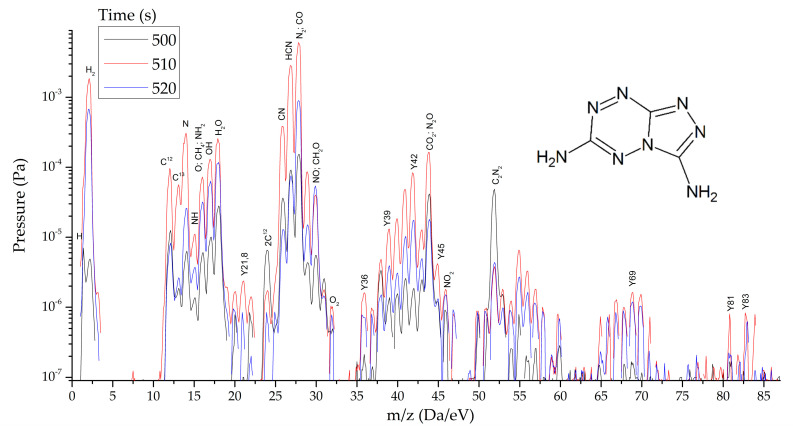
Mass spectra of gaseous products of TTDA reactions at the peak of decomposition at heating rates from 1.5 to 2000 s^−1^.

**Figure 9:**
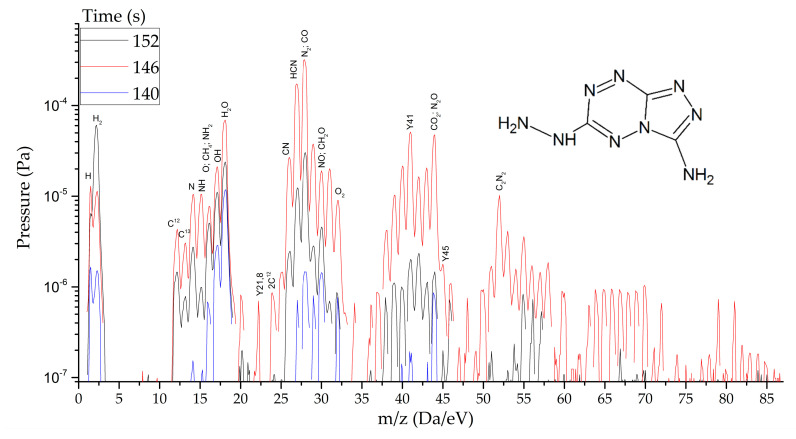
Mass spectra of gaseous products of TTGA reactions at the peak of decomposition at heating rates from 1.5 to 2000 s^−1^.

**Figure 10:**
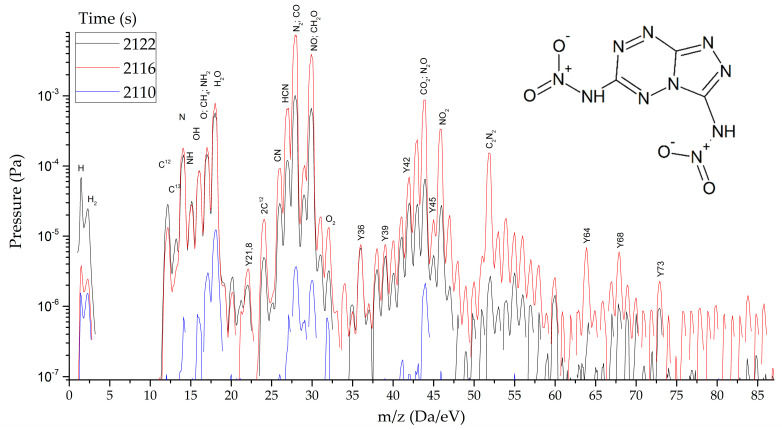
Mass spectra of gaseous products of DNTT reactions at the peak of decomposition at heating rates from 1.5 to 2000 s^−1^.

HTDMS: a sample weighing 2–3 mg was placed in a special cell located in a sealed vacuum chamber connected to the mass spectrometer channel. The procedure for pumping the gas medium to a residual pressure of 10^−5^ Pa was carried out, and the procedures for degassing the sample and registering the thermodynamic background were performed. The heating rate varied from 30 to 2000 K/s and the recorded heating range from 320 to 1200 K. The statistics of data accumulation in the mass spectrometer ranged from 50 μs/Dalton to 1 s/Dalton. The final measurement results are shown in Figure 8, Figure 9 and Figure 10.

As thermodynamic calculations and experimental measurements have shown, during the decomposition of substances of this class, the formation of gaseous nitrogen (N, N_2_), hydrogen cyanide (HCN), cyanogen (C_2_N_2_) and other substances that in some way inhibiting the decomposition process under slow exposure is observed. A similar result was obtained by the HTDMS method. In azolotetrazines containing nitro groups, there is a decrease in the concentration of hydrogen cyanide and cyane, an increase in the concentration of oxide (CO) and carbon dioxide (CO_2_), as well as nitrogen oxides (NO, NO_2_). This fact somewhat distinguishes the gas-dynamic properties of the products of explosive transformation reactions in such compounds from classical explosives.

### 2.6. Characteristics of Condensed Reaction Products after Decomposition

After the decomposition of polynitrogen compounds that do not contain a large number of nitro groups, presumably polymer carbon nitride (CN)n is formed, which according to known data [22,23,24,25,26,27,28] has a layered crystal structure similar to hexagonal graphite.

**Figure 11:**
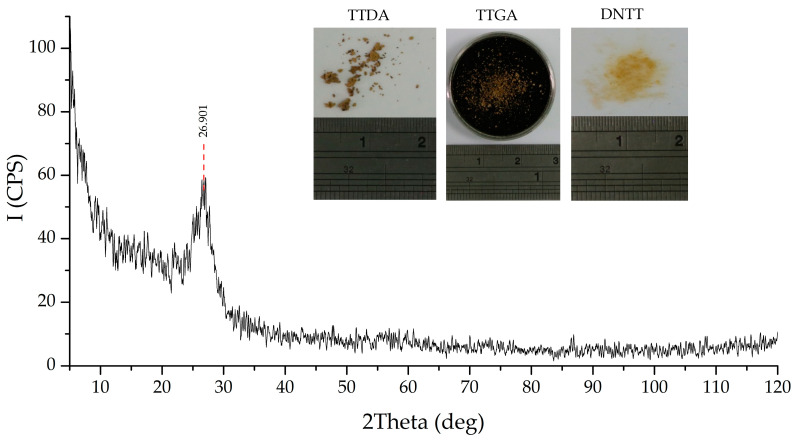
X-ray diffraction data of a powdered sample of the α-phase of carbon nitride.

The obtained results of elemental (C_15_N_23_H_3_*H_2_O), spectral, X-ray phase (Figure 11) and X-ray diffraction analysis (Table 7) confirm the assumptions made.

**Figure 12:**
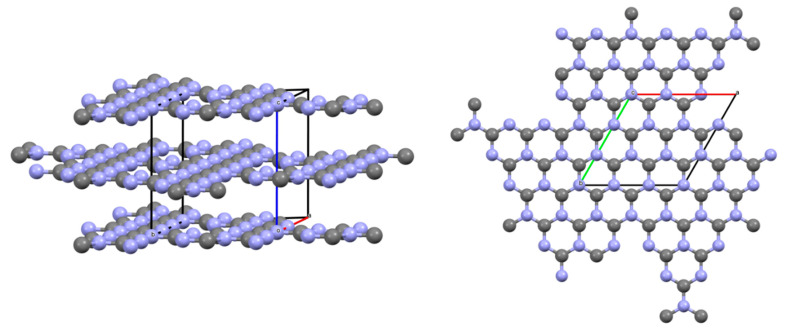
Crystallographic model of the structure of g-C_3_N_4_.

Processing of the obtained diffraction data was carried out by the method of full-profile analysis. As a result, micromorphometric characteristics were determined: the size of the coherent scattering region (crystallite), microdistorsion, condensed products of decomposition reactions of the studied azolotetrazine derivatives. The results are shown in Table 7 and Figure 12.

The formed products have a nanocrystalline structure, and the higher reaction rate results in more porous condensed carbon nitride residue. The large porosity in this case affects the color; the highly porous product has a light beige shade of brown. The product of low porosity is brown and dark brown.

Compound NyHx(CN)n absorption bands found, cm^−1^ (T%):

3573.9 (95.1); 3459.9 (93.3); 3326.3 (90.0); 3117.4 (88.6); 2883.5 (89.8); 2660.3 (91.5); 1642.8 (84.3); 1527.0 (75.5); 1404.7 (72.6); 1306.2 (71.5); 1271.0 (71.7); 1235.2 (72.6); 1155.2 (76.5); 988.2 (80.7); 856.9 (82.7); 807.9 (76.4); 776.3 (74.3); 732.1 (74.5); 544.8 (66.1).

## 3. Discussion

The analysis was carried out separately for two stages of the process:

1. Gas phase decay;
2. Solid-phase decay assisted with gas.

All calculations were performed within the recommended ICTAC [41] methods for calculating thermodynamics and kinetics based on the results of thermal analysis.

For the fundamental analysis of the kinetics of thermal decomposition and thermodynamic characteristics of the process, the methods of Kissenger [42], FWO [43,44], KAS [45], Friedman [46], Frank–Kamenetsky [47], Zhang [48,49] and Gibbs [32,33] were used.

**Figure 13:**
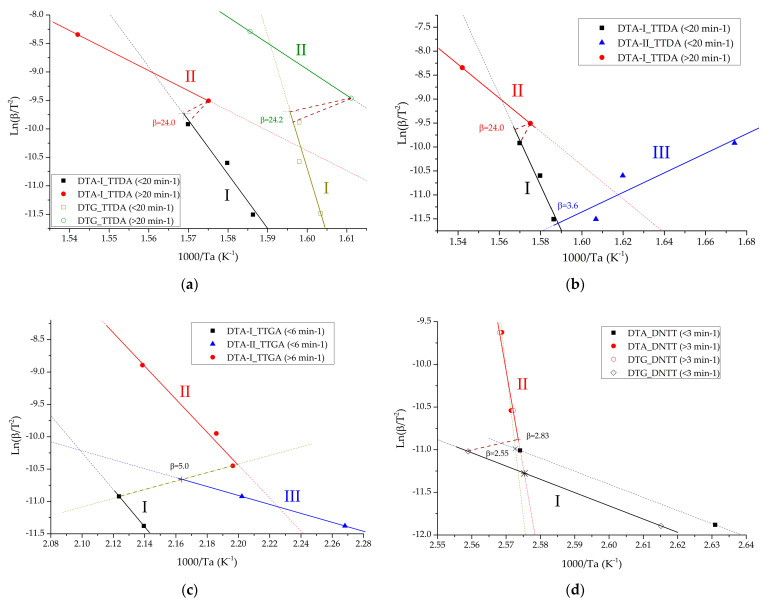
Points of change in the mechanism of chemical reactions in the studied compounds for: TTDA (**a**,**b**); TTGA (**c**) and DNTT (**d**). DTA-I-parameters of the process of intensive decomposition (maximum exothermic effect) carried out by the method of differential thermal analysis; DTG-I-parameters of the intensive decomposition process (maximum exothermic effect) carried out by thermogravimetric analysis; DTA-II-parameters of the slow decomposition process (previous exothermic effect) carried out by differential thermal analysis; DTG-II-parameters of the slow decomposition process (previous exothermic effect) carried out by thermogravimetric analysis.

Figure 13a–d show the results of determining the inflection point and changing the mechanism from slow thermal decomposition to explosive chemical reactions for the compounds under study. Formulas (9), (13) and (14) were used for the calculation.

Table 8 shows the kinetic parameters for each stage of decomposition of the studied derivatives of [1,2,4]triazolo[4,3-*b*][1,2,4,5]tetrazine.

Table 9, Table 10, Table 11, Table 12, Table 13 and Table 14 below show the results of calculating the kinetics of decomposition of the studied derivatives of [1,2,4]triazolo[4,3-*b*][1,2,4,5]tetrazine. The calculation was performed according to formulas 10–12 (chapter 4.2).

**Figure 14:**
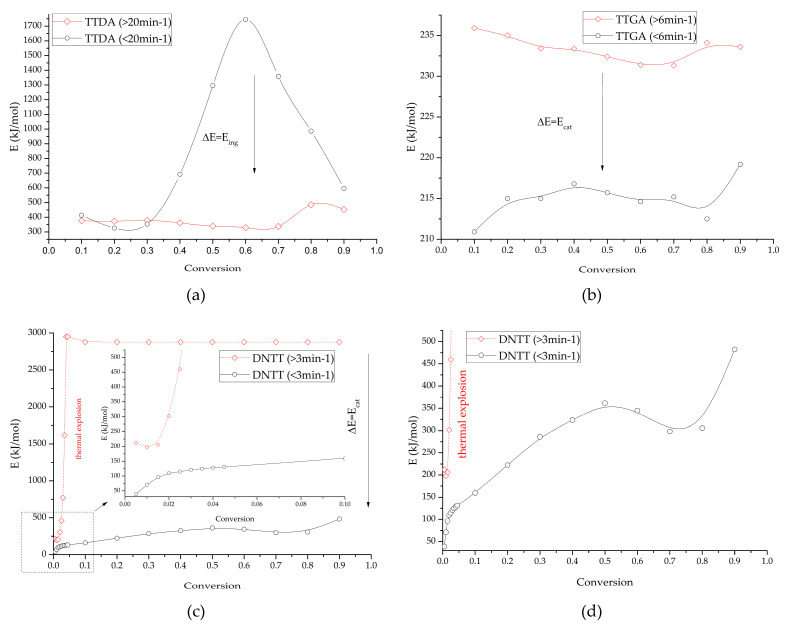
Changes in the induction energy of intensive decomposition processes depending on the catalytic contribution (taking into account gas-phase decomposition and in the absence of gas-phase decomposition) for: TTDA (**a**); TTGA (**b**) and DNTT (**c**,**d**).

**Figure 15:**
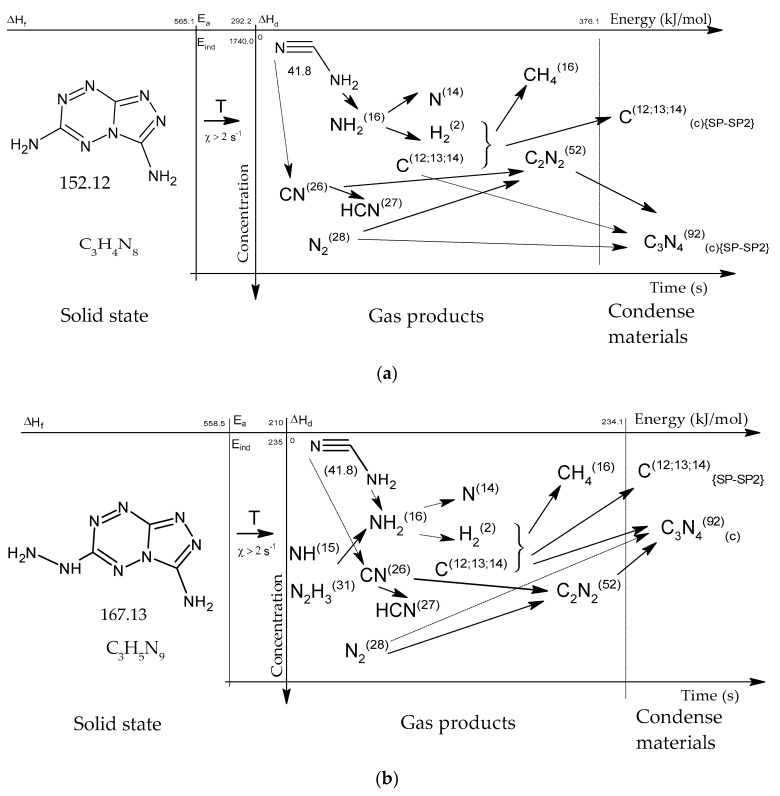

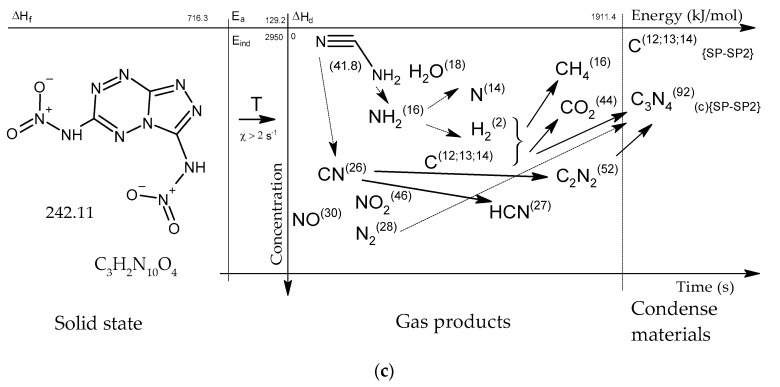
Concentration-energy diagrams of thermal decomposition reactions of [1,2,4]triazolo[4,3-*b*][1,2,4,5]tetrazine derivatives: TTDA (**a**), TTGA (**b**), DNTT (**c**) at heating rates of more than 100 min^−1^.

Figure 14 shows the inhibition and catalysis energies (autocatalysis) for the compounds under study, and Figure 15 shows the concentration-energy diagrams of reaction products obtained from calculations and experimental measurements for the compounds under study.

It is worth noting that the process of decomposition reactions is multi-stage. Two stages of decomposition are distinguishable, and in some cases three. Moreover, the lower the rate of thermal exposure, the more distinguishable are the stages of the process.

Kinetic studies have shown that the first stage of decomposition of azolotetrazine derivatives has a significantly lower rate of decomposition reactions than the second, which indicates greater stability of the base. Nevertheless, the decomposition of the base practically has an explosive character (Table 8, Table 9, Table 10, Table 11, Table 12, Table 13 and Table 14) with a transition to detonation, which negatively affects safety indicators.

The presented sequence of decomposition reactions and the decomposition scheme of [1,2,4]triazolo[4,3-*b*][1,2,4,5]tetrazine derivatives made it possible to analyze in more detail the mechanism of chemical interactions of decomposition reaction products under different effects.

Inhibition of the decomposition process occurs in the presence of up to 30 masses% of the total mass of the gas phase for each of the substances in a mixture with a solid, then a sharp explosive decomposition occurs without significant participation of inhibitors. The onset of this effect is observed with the accumulation of gases, i.e., with slow thermal exposure and thermal aging. Before the onset of the phase state under consideration, the kinetics of decomposition obey the Arrhenius law without correction for inhibitors. These, apparently, are the molecules of the substances themselves in the gas phase and cyanides. It can be assumed that due to the presence of a significant number of amino groups and nitrogen-containing fragments, the radicals of primary gas-phase decomposition bind, which is indicated not only by an increase in the temperature of thermal solid-phase decomposition, but also by the data obtained on compliance with the Arrhenius law during decomposition of this class of substances after heat treatment, as a result of which substances have a higher resistance to sublimation and the transition to the gas phase.

The results of calculations of thermal stability and autocatalysis of decomposition reactions in the studied derivatives of [1,2,4]triazolo[4,3-*b*][1,2,4,5]tetrazine obtained by Formulas (15)–(19) (chapter 4.2) are shown in Table 15.

Positive values of ΔG*, ΔH*, ΔS* of the limiting stage of decomposition indicate the absence of spontaneous reactions. Nevertheless, in each of the samples there are at least 3 points of change of the reaction mechanism.

## 4. Materials and Methods

In this paper, various methods were used to study the mechanisms for chemical decomposition of HECs, the sequence of which allowed us to exclude the non-triviality of solving the problem of directions of chemical reactions. Studies of the molecular and crystalline structure of the synthesized compounds, carried out by using nuclear magnetic resonance, time-of-flight mass spectrometry, vibrational spectroscopy and powder X-ray diffraction methods made it possible to decipher their structures without complex sample preparation operations: unfortunately, the cultivation of high-quality single crystals is not always possible. In addition, studies of the structure of powders allowed us to determine the actual chemical composition of substances used in the study of the mechanisms and kinetics of decomposition. The methods of derivatography and high-temperature dynamic mass spectrometry were used to study the kinetics of decomposition and the mechanisms of chemical interactions. In order to exclude non-triviality in the form of exothermic phase transitions, thermorentgenography methods have been applied. The mono phase of the samples has been confirmed by the X-ray phase analysis. The thermodynamic calculations made it possible to characterize the properties of reaction products in a wide range of their states, from sublimation of matter to compressed cold plasma. Comparisons of computational and theoretical concepts and experimentally measured values, characterizing the composition of reaction products and the mechanisms of their interactions, are carried out.

Analysis of the difference in the activation and induction energies of decomposition in the gas and solid phases enabled us to establish the energy of catalysis (inhibition) of the decomposition process. The use of thermorentgenography methods allows one to refute the hypothesis of an isothermal phase transition under thermal influences.

### 4.1. Synthesis and Materials Preparation

**Scheme 3:**
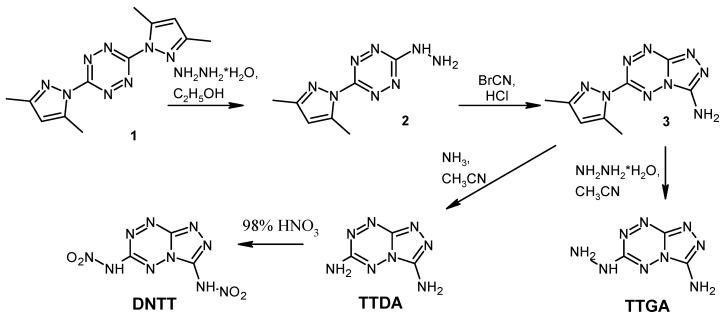
The synthesis of triazolo[4,3-*b*][1,2,4,5]tetrazine derivatives.

The synthesis of compounds was carried out according to the known methods [13,18,29] from easily available 3,6-di(3,5-dimethylpyrazole-1-yl)-1,2,4,5-tetrazine **1 [36]** (Scheme 3). Nucleophilic substitution of the 3,5-dimethylpyrazolyl moiety in tetrazine **1** on treatment with hydrazine yields compound **2**, which reacts readily with bromocyanide to form 3-amino-6-(3,5-dimethylpyrazole-1-yl)-[1,2,4]triazolo[4,3-*b*][1,2,4,5]tetrazine (**3**). Then compound **3** was modified by using the reactions with ammonia and hydrazine hydrate, leading to the formation of TTDA and TTGA, respectively. Nitration of TTDA with 98% HNO_3_ allowed us to obtain DNTT in a good yield of 91%.

The obtained materials were recrystallized and purified from impurities.

### 4.2. Thermodynamics of Decomposition Processes

In this paper, the problem of thermodynamic equilibrium was solved directly from the basic laws of thermodynamics, and the system was characterized by a maximum of entropy. The system of equations was compiled on the basis of an analytical relationship between the entropy value of the unit mass of the working fluid and the thermodynamic parameters that determine the composition and conditions of the equilibrium existence of the system [31,32].

The entropy of a multicomponent system was estimated as follows:

(1) $$S=\sum_{g=1}^{k}S_{g}^{\left(p_{g}\right)}\cdot n_{g}+\sum_{c=1}^{C}S_{c}\cdot n_{c}=\sum_{g=1}^{k}\left(S_{g}^{0}-R_{0}\ln\frac{R_{0}Tn_{g}}{\nu }\right)\cdot n_{g}+\sum_{c=1}^{C}S_{c}^{0}\cdot n_{c}$$

where *k*–number of components of the gas phase; *L*–number of condensed phases; *n_g_, n_c_*–molar content of substances, mol/kg; *S_i_^0^, S_l_^0^*–entropy of substances in the standard state, J/(mol·K); *T*–temperature, K; *v*–specific volume, m^3^/kg.

Under the accepted assumptions, the entropy of a substance in a condensed state does not depend on pressure, therefore *S_l_* = *S_l_^0^*.

Total enthalpy dH, [J/kg]. The enthalpy of the whole system is also the sum of the enthalpy of its constituent phases.

(2) $$dH=\sum_{i=1}^{k+C}I_{i}\cdot n_{i}$$

The enthalpy of individual substances is calculated from the temperature of 298.15K, accepted as standard, and includes the enthalpy of formation of substances as a measure of their chemical energy

(3) $$I_{i}=H_{i}+\Delta_{f}H_{i298}^{0}=\int_{298}^{T}C_{pi}dT+\Delta_{f}H_{i298}^{0}$$

where *C_pi_* – is the specific heat capacity of an individual substance at constant pressure, J/(mol·K).

The mass fraction of condensed phases Z is the ratio of the mass of all condensed substances to the mass of the system as a whole.

The composition of the decay products was characterized when solving the Lagrange function, in the form:

(4) $$\frac{\partial \Lambda }{\partial x_{g}}=G_{g}-R_{0}\ln\frac{R_{0}T}{\nu }-R_{0}x_{g}+\sum_{j=1}^{m}a_{jg}\lambda_{j}+a_{eg}\lambda_{e}=0,\;(g=1,\;2,\;\ldots k)$$

(5) $$\frac{\partial \Lambda }{\partial x_{c}}=\left(G_{c}+\sum_{j=1}^{m}a_{jc}\lambda_{j}\right)\cdot \exp x_{c}=0,\;(c=1,\;2,\;\ldots C)$$

where

$$G_{g}=S_{g}^{0}-\frac{U_{g}+R_{0}T}{T}=S_{g}^{0}-\frac{I_{g}}{T},\;G_{c}=S_{c}^{0}-\frac{U_{c}}{T}$$

and where G is the reduced Gibbs energy of a substance in a gaseous (*g*) and condensed (*c*) state; *I_g_* is the total enthalpy of a substance in a gaseous state.

### 4.3. Theoretical Foundations of the Analysis of Experimental Data on the Kinetics of Thermal Decomposition

The change in the proportion of the reacted substance over time during the course of chemical reactions is usually represented as:

(6) $$\frac{d\alpha }{dt}=kf\left(\alpha \right)$$

where *k* is the rate constant of chemical reactions (s^−1^); *α* is the proportion of unreacted substance; *f(α)* is the dependence of the change in the proportion of the reacted substance on time.

The kinetics of chemical reactions is given by the Arrhenius equation:

(7) $$k=Ae^{-\frac{E_{}}{RT}}$$

or

(8) $$\frac{d\alpha }{dt}=Ae^{-\frac{E}{RT}}f\left(\alpha \right)$$

where *A* is the pre-exponential multiplier, (s^−1^); *E* is the activation or adiabatic induction energy of chemical reactions (J/mol); *R* is the universal gas constant (8.314 J/mol*K); *T* is the absolute temperature of the process start (K).

To experimentally determine the rate constant of chemical reactions according to thermal analysis, we will use known methods:

1. The Kissinger method [42]

(9) $$\ln\frac{\chi }{T_{r}^{2}}=\ln\frac{AR}{E_{}g\left(x\right)}-\frac{E}{RT_{r}}$$

2. The Flynn–Wall–Ozawa (FWO) method [43,44]

(10) $$\log\left(\chi \right)=\log\frac{A_{\left(\alpha \right)}E_{\left(\alpha \right)}}{g\left(\alpha \right)R}-2.315-0.457\frac{E_{\left(\alpha \right)}}{RT_{\left(\alpha \right)}}$$

3. The Kissinger–Akahira–Sunose (KAS) method [45]

(11) $$\ln\frac{\chi }{T_{\left(\alpha \right)}^{2}}=\ln\frac{AR}{E_{}g\left(x\right)}-\frac{E_{}}{RT_{\left(\alpha \right)}}$$

4. Friedman’s method [46]
  (12) $$\ln\left(\chi \frac{d\alpha }{dT_{\left(\alpha \right)}}\right)=\ln\left[kf\left(\alpha \right)\right]-\frac{E_{\left(\alpha \right)}}{RT_{\left(\alpha \right)}}$$
  where *χ* is the rate of thermal exposure (s^−1^), E is the energy: activation or induction (J/mol).

The equilibrium points of various chemical reactions during the decomposition of the studied azolotetrazines will be searched for under the conditionk_1_ = k_2_ = k_n_. In this case, the equilibrium point of chemical reactions in Arrhenius coordinates can also be given by the ratio:

(13) $$\varpi ={\left(A^{T}A\right)}^{-1}A^{T}b$$

where *ϖ* is the point with coordinates (1/T; lnχ/T^2^); *A* is the matrix; *b* is the column vector. Having performed all the transformations with respect to the equilibrium thermal effect for the plane case, we obtain:

(14) $$T_{r}=\frac{\left(E_{1}-E_{2}\right)}{R\ln\frac{E_{2}g_{2}\left(x\right)}{E_{1}g_{1}\left(x\right)}}$$

where *E*_1_, *E*_2_ are the activation or adiabatic induction energies of the chemical reactions (J/mol), depending on the reaction mechanism.

The thermodynamic properties at the decomposition point were determined from the known ratios [47,48]:

(15) $$A\cdot \exp\frac{-E}{RT}=\nu \cdot \exp\frac{-\Delta G*}{RT}$$

(16) $$\begin{array}{l} \Delta H*=E-RT\\ \Delta G*=\Delta H*-T\Delta S*\\ \nu =\frac{k_{b}}{h}T \end{array}\}$$

where *k_b_* is the Boltzmann constant (1.38 10^−23^ J/s), *h* is Planck‘s constant (6.626 10^−34^ J/s).

Critical parameters of thermal explosion in isothermal conditions: explosion temperature (*T_m_*) and the temperature of the autocatalytic process (*T_SADT_*) are evaluated from known representations [48,49]:

(17) $$T_{m}=\frac{E_{a}-\sqrt{E_{a}^{2}-4E_{a}RT_{p0}}}{2R}$$

(18) $$T_{p0}=T_{pi}-\left(a\chi +b\chi^{2}+c\chi^{3}\right)$$

(19) $$T_{SADT}=T_{m}-\frac{RT_{m}^{2}}{E_{a}}$$

## 5. Conclusions

The processes of solid-phase decomposition of azolotetrazines with gas-phase assistance, although they obey the Arrhenius law, are largely irregular and unstable types of decomposition. Presumably, this fact is associated with the discreteness of the release of inhibitors of the decomposition of cyanogen and hydrogen cyanide, as well as the manifestation of catalytic processes when concentrations above 30 wt% are reached in the reaction zone. Moreover, lower concentrations of slow decay products lead to an increase in the heat resistance of the solid phase, which may indicate the inhibitory effect of hydrogen cyanide and cyanogen on the decomposition processes until the moment when the crystals of the substance disintegrate. The stability of the crystalline state allows us to speak of higher decomposition temperatures compared to the gas phase. Thermodynamic calculations have shown that the formation of cyanogen and hydrogen cyanide are endothermic processes, which also confirms their inhibitory effect. Characteristic of these compounds is also a sharp increase in temperature, followed by cooling by a flow of process gas-nitrogen.

Based on the results of this work, the activation energy (Ea) of the decomposition process was found for some azolotetrazine derivatives. For TTDA we found 129.0 kJ/mol, for TTGA we found 212.2 kJ/mol and for DNTT we found 292.2 kJ/mol. The average induction energy (Eind) of the catalytic process (Ecat) for the TTGA sample was 21 kJ/mol, for DNTT 1500–1700 kJ/mol. The induction energy of the inhibition process (Eing) of TTDA was 800–1400 kJ/mol.

## 6. Patents

The authors have filed an application for the invention of a method for obtaining various modifications of nanostructured carbon nitride.

## Figures and Tables

**Table 1:** The known characteristics of the studied high-energy compounds.

Sample	T_dec_ [°C]	ρ_mc_ [g/cc]	Δ_f_H_(S)_ [kJ/mol]	D [m s^−1^]	P [GPa]	IS [J]	FS [N]
TTDA		1.61	593.12	7190 [30]	21.43		
DNTT	138	1.91	740.9	9301 [2,3,13,14]	38.3	3	>5

T_dec_ [°C]-experimental decomposition temperature; ρ_mc_ [*g/cc*]- the calculated density of a single crystal obtained by quantum chemistry methods; Δ_f_H_(S)_ [kJ/mol]-calculated values of the enthalpy of formation obtained by quantum chemistry methods; P [GPa]-calculated values of the detonation pressure at the Chapman–Jouguet point; D [m s^−1^]-calculated values of detonation rates; IS [J]-impact sensitivity; FS [N]-friction sensitivity.

**Table 2:** Calculated characteristics of high-energy compounds.

Sample	Gross Formula	MW [Dalton]	ρ_mc_ [*g/cc*]	OB [%]	Δ_f_H_SE_ [kJ/mol]	V [m^3^/kg]	Z	D [m s^−1^]
TTDA	C_3_H_4_N_8_	152.121	1.592	−84.21	565.1	0.814	0.1579	7040
TTGA	C_3_H_5_N_9_	167.13	1.586	−81.44	558.5	0.852	0.1258	6960
DNTT	C_3_H_2_N_10_O_4_	242.112	1.914	−19.83	716.3	0.767	0.074	9290

MW [Dalton] – molecular weight; ρ_mc_ [*g/cc*] – the calculated density of a single crystal obtained by the group contribution method [33,34,35]; OB – oxygen balance, %; Δ_f_H_SE_ [kJ/mol] – calculated values of the enthalpy of formation obtained by semi-empirical methods of quantum chemistry; V [m^3^/kg] – calculated values of the volume of gaseous reaction products; Z – calculated values of the mass fraction of condensed residues; D [m s^−1^] – calculated values of detonation velocities obtained by the group contribution method.

**Table 3:** Crystal structure of energetic compounds.

Sample	c [%]	Ds [Å]	Symmetry Group	Unit Cell Parameter								
				*a* [Å]	*b* [Å]	*c* [Å]	α [°]	β [°]	γ [°]	Z	*ρ* [*g/cc*]	*V* [Å^3^]
TTDA	99	276	P21 21 21	10.653	8.988	6.437	90	90	90	4	1.640	616.37
TTGA	97	293	P2/m	13.620	7.97	13.230	90	97.93	90	8	1.561	1422.4
DNTT	83	316	P-1	14.669	11.775	9.412	104.89	73.94	96.56	8	1.896	1508.17

c is the degree of crystallinity [%]; a, b, c, α, β, γ, *V* are the unit cell parameters and unit cell volume; Z is the number of formula units in the unit cell; *ρ* is the density [*g/cc*]; Ds is the size of the coherent scattering region (crystallites and micro-distortions (size-strain parameters)) [Å].

**Table 4:** Decomposition points of samples according to DTA data.

Sample	β [°C/min]	Exothermic Effect-I			Exothermic Effect-II			ΣdQ [kJ/mol]
		Tsd [°C]	Tmax [°C]	Te [°C]	Tsd [°C]	Tmax [°C]	Te [°C]	
TTDA	4	332.4	339.3	-	349.7	357.2	355.9	150.43
	10	332.6 (325.9)	344.1	-	351.7	359.8	360.3	226.99
	20		324.2	345.5	346.7	363.8	378.1	255.97
	30				346 (324.8)	361.7	369.8	319.71
	100				356.6 (343.8)	375.3	417.8	407.97
TTGA	2.5	161.2	167.7	179.07	182.8	194.2	194.6	274.26
	4	165.1	172.3	179.8	183.4	197.7	197.7	316.80
	6					182.1	183.8	324.45
	10	170.3	180.9	-	179.1	184.3	194.8	335.77
	30	186.1	189.8	-	188.7	194.4	234.7	370.32
DNTT	1	96.4	106.9	116.5	118.1	124.7	133.8	354.96
	2.5	101.2	115.3	120.4	121.5	127.6	140.2	413.27
	4	110.5	115.7	116.5	-	117.6	120.5	516.34
	10	112.7	116.1	116.8	-	119.4	122.6	1567.86

**Table 5:** Decomposition points of samples according to gravimetry data II exothermic effect.

Sample	β [°C/min]	TG			DTGA		
		T_0_^s^[°C]	Tsid [°C]	Z_1(T)_ [%]	Tsd [°C]	Tsid [°C]	Te [°C]
TTDA	4	110	317	42.5		350.5	358.1
	10	175	332	47.8	240.3	352.6	368.7
	20	180	342	45.4	299.6 (348.8)	352.6	365.5
	30	180	346	50.6	283.4	347.5	367.5
	100	194	357	44.5	300.1	357.5	381.2
TTGA	2.5	165.5	191.1	14.7			
	4	168.6	195.1	14.4			
	6	176.8	178.3	12.6	178.6	179.1	181.3
	10	178.4	179.8	14.7	179.8	180.6	183.9
	30	183.3	188.9	14.1	188.8	189.7	193.4
DNTT	1	102.9		37	99.3	109.2	113.4
	2.5	109.8		41	104.8	117.6	119.7
	4	112.5	115.7	-	118.3	115.6	117.7
	10	113.5	116.2	-	115.9	116.2	118.3

T_0_^s^–start sublimation temperature, [°C]; Tsd–process start temperature, (the temperature of the beginning of decomposition by the tangent method), [°C]; Tsid–temperature of the intensive stage of the process, [°C]; Te–end temperature of the process, [°C]; Z_1(T)_–is the mass fraction of the substance at the time of completion of the self-propagating exothermic transformation at temperature T [°C], [%]; Tmax–temperature at the point of maximum decomposition rate, [°C].

**Table 6:** Experimentally determined characteristics of the studied substances.

Sample	Tsid [°C]	Tsd [°C]	Qp, J/g	Z_0_	Z_1(T)_	Z_2(T)_
TTDA	351	335	1231	0.762–0.997	0.47_(366)_	0.45_(400)_
TTGA	180	176	1961	0.988–0.998	0.142_(187)_	0.113_(250)_
DNTT	115	110	5786	0.989–1.00	0.08_(118)_ (thermal explosion)	-

Z_0_ is the mass fraction of the substance at the time of the onset of intensive thermal decomposition, at Tsid-Tsd, °C; Z_1(T)_ is the mass fraction of the substance at the time of completion of the self-propagating exothermic transformation at temperature T °C; Z_2(T)_ is the mass fraction of the condensed residue at the final temperature T, °C. Experimental data are given for a heating rate of 10 °C/min.

**Table 7:** Crystal structure of the obtained α-phase of carbon nitride.

Sample	c [%]	Ds [Å]	Symmetry Group	Cell Parameters								
				*a* [Å]	*b* [Å]	*c* [Å]	α [°]	β [°]	γ [°]	Z	*ρ* [*g/cc*]	*V* [Å^3^]
TTDA	99	96	P-6	6.694	6.694	6.649	90	90	120	2	2.652	249.45
TTGA	97	93	P1	6.723	6.723	6.948	90	90	120	2	2.550	253.37

c is the degree of crystallinity [%]; a, b, c, α, β, γ, *V* are the unit cell parameters and unit cell volume; Z is the number of formula units in the unit cell; *ρ* is the density [*g/cc*]; Ds is the size of the coherent scattering region (crystallites and micro-distortions (size-strain parameters)) [Å].

**Table 8:** Kinetic parameters of the limiting stage of thermal decomposition in the solid phase obtained by the Kissenger method.

Sample	Stage	Kinetic Parameters According to the Data					
		DTA/DSC			DTGA		
		E [kJ·mol^−1^]	A [s^−1^]	R^2^	E [kJ·mol^−1^]	A [s^−1^]	R^2^
TTDA	I (cat)	784.0	1.63 × 10^60^	0.96	1939.6	1.4 × 10^158^	0.81
	II	292.2	4.91 × 10^19^	1.0	381.3	7.51 × 10^27^	1.0
	III (ing)	169.4	2.70 × 10^−20^	0.83			
TTGA	I	237.7	2.11 × 10^21^	1.0	216.5	1.04 × 10^19^	1.0
	II	212.2	3.09 × 10^19^	0.98	238.0	5.04 × 10^22^	0.96
	III	57.4	8.28	1.0			
DNTT	I				129.0	7.56 × 10^11^	1.0
	II	2170	7.6 × 10^287^	0.966	1914	2.4 × 10^253^	1.0

**Table 9:** Kinetic parameters of TTDA at heating rates less than 20 min^−1^.

Conversion	FWO		KAS		Friedman	
	E [kJ·mol^−1^]	A [s^−1^]	E [kJ·mol^−1^]	A [s^−1^]	E [kJ·mol^−1^]	A [s^−1^]
0.1	404.5	3.4 × 10^17^	409.3	6.0 × 10^31^	414.2	9.7 × 10^33^
0.2	319.8	4.0 × 10^13^	321.3	6.7 × 10^22^	326.4	2.2 × 10^25^
0.3	346.4	2.9 × 10^14^	348.6	5.3 × 10^24^	353.8	2.7 × 10^27^
0.4	673.2	1.0 × 10^27^	687.2	1.8 × 10^53^	692.4	1.2 × 10^56^
0.5	1254.7	1.1 × 10^49^	1289.7	4.5 × 10^103^	1294.9	3.8 × 10^106^
0.6	1689.3	2.9 × 10^65^	1740.0	2.0 × 10^141^	1745.2	2.0 × 10^144^
0.7	1315.9	1.9 × 10^51^	1353.1	5.8 × 10^108^	1358.3	7.0 × 10^111^
0.8	956.5	3.6 × 10^37^	980.7	2.2 × 10^77^	985.9	3.0 × 10^80^
0.9	579.8	1.2 × 10^23^	590.3	2.1 × 10^44^	595.5	3.2 × 10^47^
Mean	837.8		857.8		863.0	

**Table 10:** Kinetic parameters of TTDA at heating speeds of more than 20 min^−1^.

Conversion	FWO		KAS		Friedman	
	E [kJ·mol^−1^]	A [s^−1^]	E [kJ·mol^−1^]	A [s^−1^]	E [kJ·mol^−1^]	A [s^−1^]
0.1	368.6	6.4 × 10^15^	371.8	6.4 × 10^27^	376.9	1.1 × 10^30^
0.2	365.0	2.4 × 10^15^	367.9	6.7 × 10^26^	373.1	2.3 × 10^29^
0.3	370.8	3.7 × 10^15^	373.8	1.7 × 10^27^	379.0	8.9 × 10^29^
0.4	354.0	8.0 × 10^14^	356.4	5.5 × 10^25^	361.6	3.8 × 10^28^
0.5	333.2	1.2 × 10^14^	334.8	7.4 × 10^23^	340.0	6.4 × 10^26^
0.6	322.9	4.6 × 10^13^	324.1	8.4 × 10^22^	329.3	8.7 × 10^25^
0.7	330.4	8.3 × 10^13^	331.9	3.1 × 10^23^	337.2	3.8 × 10^26^
0.8	473.1	2.0 × 10^19^	479.7	4.7 × 10^35^	485.0	6.5 × 10^38^
0.9	442.6	1.0 × 10^18^	448.0	5.5 × 10^32^	453.3	8.7 × 10^35^
Mean	373.4		376.5		381.7	

**Table 11:** Kinetic parameters of TTGA at heating rates less than 6 min^−1^.

Conversion	FWO		KAS		Friedman	
	E [kJ·mol^−1^]	A [s^−1^]	E [kJ·mol^−1^]	A [s^−1^]	E [kJ·mol^−1^]	A [s^−1^]
0.1	211.1	3.0 × 10^11^	210.9	2.4 × 10^18^	214.8	3.1 × 10^20^
0.2	215.0	4.7 × 10^11^	215.0	6.8 × 10^18^	218.9	1.8 × 10^21^
0.3	215.0	4.7 × 10^11^	215.0	6.9 × 10^18^	218.9	2.7 × 10^21^
0.4	216.7	5.8 × 10^11^	216.8	1.1 × 10^19^	220.7	5.7 × 10^21^
0.5	215.6	5.1 × 10^11^	215.7	8.1 × 10^18^	219.5	5.2 × 10^21^
0.6	214.6	4.5 × 10^11^	214.6	6.0 × 10^18^	218.4	4.7 × 10^21^
0.7	215.2	4.8 × 10^11^	215.2	7.1 × 10^18^	219.1	6.4 × 10^21^
0.8	212.6	3.5 × 10^11^	212.5	3.4 × 10^18^	216.4	3.5 × 10^21^
0.9	219.0	7.4 × 10^11^	219.2	1.9 × 10^19^	223.1	2.2 × 10^22^
Mean	215.0		215.0		218.9	

**Table 12:** Kinetic parameters of TTGA at heating rates of more than 6 min^−1^.

Conversion	FWO		KAS		Friedman	
	E [kJ·mol^−1^]	A [s^−1^]	E [kJ·mol^−1^]	A [s^−1^]	E [kJ·mol^−1^]	A [s^−1^]
0.1	235.1	1.8 × 10^13^	235.9	2.8 × 10^22^	239.8	3.5 × 10^24^
0.2	234.2	1.6 × 10^13^	235.0	2.1 × 10^22^	238.8	5.4 × 10^24^
0.3	232.6	1.3 × 10^13^	233.4	1.4 × 10^22^	237.2	5.3 × 10^24^
0.4	232.6	1.3 × 10^13^	233.4	1.4 × 10^22^	237.2	7.0 × 10^24^
0.5	231.6	1.2 × 10^13^	232.4	1.0 × 10^22^	236.2	6.5 × 10^24^
0.6	230.7	1.0 × 10^13^	231.4	7.9 × 10^21^	235.2	6.0 × 10^24^
0.7	230.6	1.0 × 10^13^	231.3	7.5 × 10^21^	235.1	6.7 × 10^24^
0.8	233.3	1.4 × 10^13^	234.1	1.6 × 10^22^	238.0	1.6 × 10^25^
0.9	232.8	1.3 × 10^13^	233.6	1.3 × 10^22^	237.4	1.4 × 10^25^
Mean	232.6		233.4		237.2	

**Table 13:** Kinetic parameters of DNTT at heating rates less than 3 min^−1^.

Conversion	FWO		KAS		Friedman	
	E [kJ·mol^−1^]	A [s^−1^]	E [kJ·mol^−1^]	A [s^−1^]	E [kJ·mol^−1^]	A [s^−1^]
0.005	43.4	4.3 × 10^3^	38.9	2.6 × 10^1^	41.9	1.3 × 10^2^
0.01	74.4	6.2 × 10^5^	71.0	1.2 × 10^6^	74.0	1.2 × 10^7^
0.015	98.8	2.5 × 10^7^	96.2	4.3 × 10^9^	99.3	6.7 × 10^10^
0.02	111.9	1.8 × 10^8^	109.8	3.2 × 10^11^	112.9	6.6 × 10^12^
0.025	116.7	3.4 × 10^8^	114.7	1.3 × 10^12^	117.8	3.5 × 10^13^
0.03	123.0	8.4 × 10^8^	121.1	9.9 × 10^12^	124.3	3.1 × 10^14^
0.035	126.3	1.3 × 10^9^	124.6	2.7 × 10^13^	127.7	1.0 × 10^15^
0.04	129.8	2.2 × 10^9^	128.2	8.1 × 10^13^	131.3	3.4 × 10^15^
0.045	132.4	3.1 × 10^9^	130.9	1.8 × 10^14^	134.0	8.6 × 10^15^
0.1	159.9	2.4 × 10^10^	159.3	1.5 × 10^16^	162.5	1.6 × 10^18^
0.2	220.8	1.0 × 10^14^	222.3	2.2 × 10^24^	225.5	4.7 × 10^26^
0.3	282.4	3.8 × 10^17^	286.0	2.6 × 10^32^	289.3	8.6 × 10^34^
0.4	318.8	4.1 × 10^19^	323.7	1.1 × 10^37^	327.0	4.7 × 10^39^
0.5	355.0	3.5 × 10^21^	361.1	2.6 × 10^41^	364.5	1.4 × 10^44^
0.6	339.3	1.7 × 10^20^	344.7	2.5 × 10^38^	348.2	1.7 × 10^41^
0.7	294.5	2.3 × 10^17^	298.1	6.9 × 10^31^	301.6	5.5 × 10^34^
0.8	301.5	3.7 × 10^17^	305.3	1.9 × 10^32^	308.9	1.8 × 10^35^
0.9	472.3	6.3 × 10^26^	482.2	1.9 × 10^53^	485.8	2.1 × 10^56^

**Table 14:** Kinetic parameters of DNTT at heating rates of more than 3 min^−1^.

Conversion	FWO		KAS		Friedman	
	E [kJ·mol^−1^]	A [s^−1^]	E [kJ·mol^−1^]	A [s^−1^]	E [kJ·mol^−1^]	A [s^−1^]
0.005	210.7	6.3 × 10^14^	212.0	1.7 × 10^26^	215.1	8.7 × 10^26^
0.01	196.7	5.7 × 10^13^	197.4	7.1 × 10^23^	200.6	7.5 × 10^24^
0.015	205.0	1.6 × 10^14^	206.0	7.2 × 10^24^	209.2	1.2 × 10^26^
0.02	297.4	9.0 × 10^19^	301.7	7.6 × 10^37^	305.0	1.6 × 10^39^
0.025	450.0	2.2 × 10^29^	459.8	1.9 × 10^59^	463.1	5.1 × 10^60^
0.03	749.9	4.8 × 10^47^	770.5	1.6 × 10^101^	773.8	5.2 × 10^102^
0.035	1566.2	2.1 × 10^97^	1616.4	1.2 × 10^215^	1619.6	4.4 × 10^216^
0.04	2853.7	2.2 × 10^175^	2950.4	1.92 × 10^394^	2953.7	2.86 × 10^396^
0.045	2853.7	2.2 × 10^175^	2950.4	1.92 × 10^394^	2953.7	2.86 × 10^396^
0.1…0.9	2782.7	1.1 × 10^171^	2876.9	1.92 × 10^384^	2880.1	7.77 × 10^386^

**Table 15:** Thermodynamic properties of the decomposition process in DTA cell.

Sample	T_SADT_	Tm	ΔG*	ΔH*	ΔS*
	[°C]	[°C]	[kJ·mol^−1^]	[kJ·mol^−1^]	[J·mol^−1^·K^−1^]
TTDA	349.5	358.2	183.3	376.1	308.3
TTGA	190.3	198.0	135.9	234.1	211.8
DNTT	113.4	114.1	121.4	1911.4	4629.4

ΔG*, ΔH*, ΔS* are the Gibbs energy, enthalpy, and the entropy of the decomposition process respectively; T_SADT_ is the temperature of the beginning of the autocatalytic decomposition process; Tm is the thermal explosion temperature.

## Data Availability

Not applicable.

## Sample Availability

Samples of the compounds are available from the authors.

## References

[1] Luo Y., Zheng W., Wang X., Shen F. (2022). Nitrification Progress of Nitrogen-Rich Heterocyclic Energetic Compounds: A Review. Molecules.

[2] Gao H., Zhang Q., Shreeve J.M. (2020). Fused heterocycle-based energetic materials (2012–2019). J. Mater. Chem. A.

[3] Herweyer D., Brusso J.L., Murugesu M. (2021). Modern trends in “Green” primary energetic materials. New J. Chem..

[4] Pagoria P.F., Lee G.S., Mitchell A.R., Schmidt R.D. (2002). A review of energetic materials synthesis. Thermochim. Acta.

[5] Steinhauser G., Klapotke T.M. (2008). “Green” Pyrotechnics: A Chemists’ Challenge. Angew. Chem. Int. Ed..

[6] Cao W.L., Li Z.M., Yang J.Q., Zhang J.G. (2022). Recent advances on the nitrogen-rich 1,2,4-oxadiazole-azoles-based energetic materials. Def. Technol..

[7] Ostrovskii V.A., Pevzner M.S., Kofman T.P., Shcherbinin M.B., Tselinskii I.V., Attanasi O., Spinelli D. (1999). Energetic 1,2,4-triazoles and tetrazoles synthesis, structure and properties. Targets in Heterocyclic Systems: Chemistry and Properties.

[8] Shreeve J.M., Gao H., Wang T. (2021). Functionalized Tetrazole Energetics: A Route to Enhanced Performance. Z. FürAnorg. Und Allg..

[9] Tang J., Yang P., Yang H., Xiong H., Hu W., Cheng G. (2020). A simple and efficient method to synthesize high-nitrogen compounds: Incorporation of tetrazole derivatives with N5 chains. Chem. Eng. J..

[10] Wozniak D.R., Piercey D.G. (2020). Review of the current synthesis and properties of energetic pentazolate and derivatives thereof. Engineering.

[11] Chavez D.E. (2017). Energetic heterocyclic N-oxides. Heterocyclic N-Oxides.

[12] Chavez D.E., Hiskey M.A. (1999). 1,2,4,5-Tetrazine based energetic materials. J. EnergeticMater..

[13] Hu L., Yin P., Zhao G., He C., Imler G.H., Parrish D.A., Gao H., Shreeve J.M. (2018). Conjugated Energetic Salts Based on Fused Rings: Insensitive and Highly Dense Materials. J. Am. Chem. Soc..

[14] Hu L., He C., Zhao G., Imler G.H., Parrish D.A., Shreeve J.M. (2020). Selecting suitable substituents for energetic materials based on a fused triazolo[1,2,4,5]tetrazine ring. ACS Appl. Energy Mater..

[15] Hu L., Yin P., Imler G.H., Parrish D.A., Gao H., Shreeve J.M. (2019). Fused rings with N-oxide and–NH2: Good combination for high density and low sensitivity energetic materials. Chem. Commun..

[16] Konkova T.S., Matyushin Y.N., Miroshnichenko E.A., Vorobev A.B., Palysaeva N.V., Sheremetev A.B. (2020). Thermochemical Properties of [1,2,4]Triazolo[4,3-b][1,2,4,5]tetrazine Derivatives. Russ. J. Phys. Chem. B.

[17] Sinditskii V.P., Burzhava A.V., Usuntsinova A.V., Egorshev V.Y., Palysaeva N.V., Suponitsky K.Y., Ananiev I.V., Sheremetev A.B. (2020). Increasing the burning rate through energetic compound tuning: Hybrids of the furazan and [1,2,4]triazolo[4,3-*b*][1,2,4,5]tetrazine ring systems. Combust. Flame.

[18] Liu Y., Zhao G., Tang Y., Zhang J., Hu L., Imler G.H., Parrish D.A., Shreeve J.M. (2019). Multipurpose [1,2,4]triazolo[4,3-b][1,2,4,5]tetrazine-based energetic materials. J. Mater. Chem. A.

[19] Yu Q., Singh J., Staples R.J., Shreeve J.M. (2022). Assembling Nitrogen-rich, thermally Stable, and insensitive energetic materials by polycyclization. Chem. Eng. J..

[20] Chen X., Guo Z., Zhang C., Zhang J., Ma H. (2021). Boosting intermolecular interactions of fused cyclic explosives: The way to thermostable and insensitive energetic materials with high density. New J. Chem..

[21] Wang G., Lu T., Fan G., Yin H., Chen F.X. (2019). Synthesis and properties of insensitive [1,2,4]triazolo[4,3-b]-1,2,4,5-tetrazine explosives. New J. Chem..

[22] Lotsch B.V., Schnick W. (2006). From Triazines to Heptazines: Novel Nonmetal Tricyanomelaminates as Precursors for Graphitic Carbon Nitride Materials. Chem. Mater..

[23] Korsunsky B.L., Pepekin V.I. (1997). On the way to carbon nitride. Successes Chem..

[24] Thomas A., Fischer A., Goettmann F., Antonietti M., Müller J.O., Schlögl R., Carlsson J.M. (2008). Graphitic carbon nitride materials: Variation of structure and morphology and their use as metal-free catalysts. J. Mater. Chem..

[25] Tyborski T., Merschjann C., Orthmann S., Yang F., Lux-Steiner M., Schedel-Niedrig T. (2013). Crystal structure of polymeric carbon nitride and the determination of its process-temperature-induced modifications. J. Phys. Condens. Matter.

[26] Matsumoto S., Xie E.-Q., Izumi F. (1999). On the validity of the formation of crystalline carbon nitrides, C3N4. Diam. Relat. Mater..

[27] Sun B., Yu H., Yang Y., Li H., Zhai C., Qian D., Chen M. (2017). New Complete Assignments of X-ray Powder Diffraction Patterns in Graphitic Carbon Nitride Using Discrete Fourier Transform and Direct Experimental Evidence. R. Soc. Chem. Phys. Chem. Chem. Phys..

[28] Axet M.R., Durand J., Gouygou M., Chapter P.S. (2019). Two—Surface coordination chemistry on graphene and two-dimensional carbon materials for well-defined single atom supported catalysts. Adv. Organomet. Chem..

[29] Chavez D.E., Hiskey M.A. (1998). Synthesis of the bi-heterocyclic parent ring system 1,2,4-triazolo[4,3-*b*][1,2,4,5]tetrazine and some 3,6-disubstituted derivatives. J. Heterocycl. Chem..

[30] Wei T., Zhu W., Zhang J., Xiao H. (2010). DFT study on energetic tetrazolo-[1,5-b]-1,2,4,5-tetrazine and 1,2,4-triazolo-[4,3-b]-1,2,4,5-tetrazine derivatives. J. Hazard. Mater..

[31] Huff V.M., Gordon S., Morrell V.E. General Method and Thermodynamical Tables for Computing Equilibria. NACA TR-1037, 1950. https://ntrs.nasa.gov/citations/19930091090..

[32] Prigogine I. (1962). Non-Equilibrium Statistical Mechanics. Monographs in Statistical Physics. Volume 1..

[33] Dzyabchenko A.V., Pivina T.S., Arnautova E.A. (1996). Prediction of structure and density for organic nitramines. J. Mol. Struct..

[34] Tarver C.M. (1979). Density estimations for explosives and related compounds using the group additives approach. J. Chem. Eng. Data.

[35] Kotomin A.A., Kozlov A.S. (2006). Calculation of Densities of Organic Compounds from Contributions of Molecular Fragments. Russ. J. Appl. Chem..

[36] Coburn M.D., Buntain G.A., Harris B.W., Hiskey M.A., Lee K.Y., Ott D.G. (1991). An improved synthesis of 3,6-diamino-1,2,4,5-tetrazine. II. From triaminoguanidine and 2, 4-pentanedione. J. Heterocycl. Chem..

[37] Le Bail A. (1989). Structure determination of NaPbFe2F9 by X-ray powder diffraction. J. Solid State Chem..

[38] Pawley G.S. (1981). Unit-cell refinement from powder diffraction scans. J. Appl. Crystallogr..

[39] Boultif A., Louer D. (1991). Indexing of powder diffraction patterns for low-symmetry lattices by the successive dichotomy method. J. Appl. Cryst..

[40] Werner P.-E., Eriksson L., Westdahl M. (1985). TREOR, a Semi-Exhaustive Trial-and-Error Powder Indexing Program for All Symmetries. J. Appl. Cryst..

[41] Vyazovkin S., Burnham A., Criado J., Pérez-Maqueda L., Popescu C., Sbirrazzuoli N. (2011). ICTAC Kinetics Committee Recommendations for Performing Kinetic Computations on Thermal Analysis Data. Thermochim. Acta.

[42] Kissinger H.E. (1957). Reaction Kinetics in Differential Thermal Analysis. J. Therm. Anal. Chem..

[43] Ozawa T. (1965). A New Method of Analyzing Thermogravimetric Data. Bull. Chem. Soc. Jpn..

[44] Flynn J.H., Wall L. (1966). A General Treatment of the Thermogravimetry of Polymers. J. Res. Natl. Bur. Std. A Phys. Chem..

[45] Akahira T., Sunose T. (1971). Method of Determining Activation Deterioration Constant of Electrical Insulating Materials. Res. Rep. Chiba Inst. Technol. Sci. Technol..

[46] Friedman H. (1964). Kinetics of Thermal Degradation of Char-Forming Plastics from Thermogravimetry: Application to a Phenolic Plastic. J. Polym. Sci. Part C.

[47] Frank-Kamenetsky D.A. (2008). Fundamentals of Macrokinetics. Diffusion and Heat Transfer in Chemical Kinetics.

[48] Tompa A.S., Boswell R.F. (2000). Thermal Stability of a Plastic Bonded Explosive. Thermochim. Acta.

[49] Zhang T.L., Hu R.Z., Xie Y., Li F.P. (1994). The Estimation of Critical Temperatures of Thermal Explosion for Energetic Materials Using Non-isothermal DSC. Thermochim. Acta.

